# Impact of a tropical monsoon climate on formaldehyde exposure and microbial contamination in anatomy dissection hall

**DOI:** 10.1371/journal.pone.0337238

**Published:** 2025-11-21

**Authors:** Areeya Madsusan, Saowaluk Krainara, Wantanasak Suksong, Kittithat Sudchoo, Nadeyah Tohmoh, Pattharaporn Jonggrijug, Chomkaeo Maipunklang, Chanitsara Chadaram, Kholeeyoh Samaeng, Piyadhida Kurdthongmee, Uratit Noosab, Arun Nakapong, Yanawut Udomsri, Suttiporn Kanaso, Natee Sakorn, Ng Yee Guan, Sukrit Sangkhano

**Affiliations:** 1 Department of Occupational Health and Safety, School of Public Health, Walailak University, Nakhon Si Thammarat, Thailand; 2 Excellence Center for Public Health Research: EC for PHR, School of Public Health, Walailak University, Nakhon Si Thammarat, Thailand; 3 Department of Environmental Health and Technology, School of Public Health, Walailak University, Nakhon Si Thammarat, Thailand; 4 Biomass and Oil Palm Center of Excellence, Walailak University, Nakhon Si Thammarat, Thailand; 5 The Center for Scientific and Technological Equipment, Walailak University, Nakhon Si Thammarat, Thailand; 6 Faculty of Medicine and Health Science, Universiti Putra Malaysia, Selangor, Malaysia; 7 Preclinical Science Unit, School of Public Health, Walailak University, Nakhon Si Thammarat, Thailand; SKUMS: Shahrekord University of Medical Science, IRAN, ISLAMIC REPUBLIC OF

## Abstract

Gross anatomy dissection is an essential component of medical and health science education, yet it presents notable occupational hazards, particularly from formaldehyde (FA) exposure and microbial contamination. These risks may be intensified in anatomy dissection halls located in tropical monsoon (Am) climates, where elevated humidity and temperature promote both chemical volatility and microbial persistence. This study assessed the combined effects of such climatic conditions on FA concentrations and microbial ecology within a naturally ventilated dissection hall in southern Thailand. FA levels were measured through personal and area air sampling across seven anatomical regions, while microbial contamination on cadaver-contact surfaces was evaluated using culture-based methods and high-throughput sequencing. Functional prediction of microbial communities was performed using PICRUSt2 to assess their metabolic adaptation to environmental stressors. The results revealed that both personal and indoor FA concentrations (mean 1.17 ± 0.39 ppm and 1.09 ± 0.45 ppm, respectively) exceeded several international occupational exposure limits, with the highest levels observed during dissections involving deep or adipose-rich anatomical regions. Microbial analyses identified stress-tolerant and potentially pathogenic genera, including *Bdellovibrio*, *Aequorivita*, and *Aspergillus spp.*, along with enriched pathways involved in aromatic compound degradation and environmental resilience. These findings highlight the limitations of natural ventilation in controlling occupational exposures and microbial contamination in Am climate anatomy laboratories. The study supports the implementation of climate-responsive engineering controls and laboratory management strategies that address chemical safety, thermal regulation, and biosafety to promote healthier and more sustainable dissection environments in similar high-risk settings.

## Introduction

Gross anatomy dissection is vital in medical education but carries health risks, especially from formaldehyde (FA) exposure. Classified by the International Agency for Research on Cancer (IARC), as a Group 1 carcinogen, FA is a known irritant linked to respiratory distress, mucosal irritation, and cancer [1]. Previous studies have shown that FA levels in anatomy dissection halls often exceed safety thresholds, particularly where natural ventilation is used [2,3]. However, few studies have accounted for how regional climate conditions, particularly in tropical zones, may exacerbate these risks.

Anatomy dissection halls located in tropical monsoon (Am) climates, characterized by high humidity, elevated temperatures, and minimal dry periods, present unique challenges related to chemical exposure and biological contamination. The high humidity levels in these environments can accelerate FA volatilization, compromising air quality and increasing health risks for humans [4]. Additionally, excessive moisture can reduce the efficiency of natural ventilation systems, leading to the accumulation of airborne contaminants [5]. While FA is a fixative and antimicrobial agent, studies have demonstrated that formalin-fixed cadavers may still harbor viable bacteria and fungi, some of which are capable of airborne transmission and colonization of cadaver surfaces [6,7]. Furthermore, warm and humid conditions create a favorable environment for microbial proliferation, both in the air and on cadaver surfaces, potentially impacting occupational safety and specimen preservation. Indoor air in such environments often contains bioaerosols that can settle on cadavers and grow despite prior chemical fixation [8]. Cadavers preserved for anatomical education can harbor a wide range of pathogenic microorganisms, including antibiotic-resistant and opportunistic species, particularly in hot and humid climates that promote microbial persistence. Despite formalin embalming, contamination of cadaver surfaces and dissection hall air remains common. Previous studies have isolated bacteria such as *Bacillus subtilis* and fungi including *Aspergillus flavus*, *Penicillium sp*., and *Cladosporium sp*., highlighting the limited antimicrobial efficacy of preservation alone [7,8]. In southern Thailand, Nunkaew et al. [9] identified seven fungal genera on formalin-fixed cadavers, most notably *Cladosporium* and *Aspergillus* and demonstrated that routine antifungal disinfection was ineffective under high-humidity conditions. Supporting this, Ndyamuhakyi et al. [10] reported microbial colonization in 27% of cadavers used in Ugandan medical schools, with over 60% of contaminated bodies harboring both bacteria and fungi. Detected organisms included *Aspergillus*, *Penicillium*, *Alternaria*, *Blastomyces*, *Neoscytalidium dimidiatum*, and *Exophiala dermatitidis*, as well as *Staphylococcus*, *Streptococcus*, *Bacillus*, and *Streptobacillus*. While previous studies confirm microbial contamination on formalin-fixed cadavers in humid environments, they do not explain how these microbes survive. This study addresses that gap by combining environmental sampling with microbial profiling and functional gene prediction, revealing biochemical adaptations to chemically and climatically stressed conditions. The findings highlight the need for improved disinfection and climate-adaptive biosafety measures in anatomy education.

Despite these concerns, few studies have simultaneously examined both FA exposure and microbial contamination in anatomy labs located in Am climates. This study addresses that gap by investigating a gross anatomy dissection hall in southern Thailand, a region characterized by the Am climate. FA concentrations were measured through personal and indoor sampling, while microbial contamination was assessed on cadaver-contact surfaces and surrounding environments under typical teaching conditions with natural ventilation. The findings will contribute to climate sensitive laboratory design, improved ventilation strategies, and enhanced occupational health protections in tropical academic settings, where these challenges are most pronounced yet remain largely unaddressed.

## Methodology

### Study setting and experimental layout

This cross-sectional observational study was conducted between 01/08/2023–29/09/2023 in the Anatomy Hall of Walailak University located in Nakhon Si Thammarat province, southern Thailand, which has a tropical monsoon (Am) climate. Ethical approval was obtained from the Walailak University Ethics Committee (Approval No: WUEC-23-156-01), in accordance with the Declaration of Helsinki. All participants received detailed information about the study and provided written informed consent. Data were anonymized prior to analysis to ensure confidentiality and adherence to ethical standards.

Twelve embalmed cadavers were obtained from the Center for Scientific and Technological Equipment and placed individually on stainless-steel dissection tables. These tables were systematically arranged in a grid layout of three rows with four tables each, centrally located within the dissection hall. The room measured 35 meters in length, 20 meters in width, and 4.2 meters in height, totaling approximately 2,940 cubic meters of volume. The hall is situated on the second floor, about four meters above ground level.

**Fig 1:**
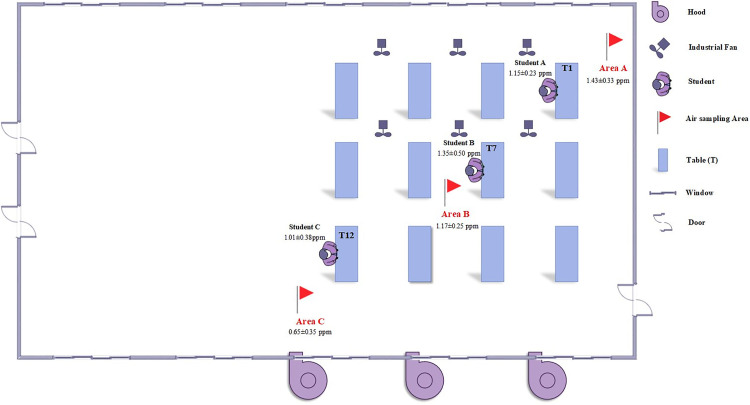
Layout of the anatomy dissection hall, sampling locations, and FA exposure levels.

To support airflow during dissection classes, the hall featured 28 windows and 3 doors along opposite walls, all kept open during sessions. Ventilation was supplemented by eight 24-inch industrial standing fans arranged in two parallel rows and three exhaust systems installed at the lower third of one side wall. This combination of natural and mechanical ventilation was intended to facilitate air exchange during cadaver-based teaching activities (Fig 1). This setting provided a representative environment to evaluate the influence of architectural design and natural ventilation on FA dispersion and microbial persistence in a climate-sensitive anatomy education context.

### Assessment of FA levels via personal and indoor air sampling

FA concentrations within the dissection hall were systematically evaluated using both personal exposure monitoring and area air sampling techniques. Personal exposure assessments were conducted on one student per cadaver table, with three tables selected to represent distinct spatial zones in the room: the front corner (Student A), center (Student B), and rear inner corner (Student C), as illustrated in Fig 1. To reduce sampling bias and improve the representativeness of the data, one student from each table was randomly selected during each dissection session, resulting in a total of 21 personal air samples collected across seven anatomical dissection topics. Selected students were equipped with calibrated personal air sampling pumps connected to silica gel-coated solid sorbent tubes, positioned at the breathing zone by attaching the equipment to the upper chest region of their gowns. Sampling was carried out continuously throughout each four-hour dissection class in accordance with the National Institute for Occupational Safety and Health Method (NIOSH) 2016, ensuring standardized and accurate assessment of Eight-hour time-weighted average (TWA) FA exposure.

In parallel, indoor air concentrations were assessed via area sampling at three fixed locations within the dissection hall. Sampling points were positioned to capture spatial variation in air quality: Area A and Area C were located at diagonally opposite corners of the room, while Area B was placed at the central region. Each sampling device was mounted on a tripod at a height of 1.5 meters from the floor and 1 meter from the nearest dissection table to simulate breathing-zone conditions in the standing position.

Personal and area air sampling were conducted over seven standardized dissection sessions, each aligned with a distinct anatomical region: Pectoral and Scapular Region (PSR), Bisection of Head and Air Sinuses (BHAS), Respiratory Tract and Lung (RTL), Oral Cavity and Pharynx (OCP), Anterior Abdominal Wall and Peritoneal Cavity (ABPP), Abdominal Viscera and Gastrointestinal Tract (AbGI), and Gluteal and Thigh Regions (GTR). Each session lasted approximately four hours, consistent with routine gross anatomy laboratory periods.

To evaluate the influence of anatomical depth on FA exposure, dissection regions were categorized into three depth levels based on procedural invasiveness and degree of internal exposure: superficial (PSR), intermediate (BHAS, RTL, OCP), and deep (ABPP, AbGI, GTR). Depth scores were assigned ordinally (1–3) for statistical analysis. This classification was informed by prior evidence demonstrating that deeper dissections, particularly those involving body cavities or adipose-rich tissues, are associated with elevated FA concentrations due to increased chemical retention and reduced ventilation efficiency [11–13].

### FA sample analysis

The methodology for all sampling FA in both personal and indoor sampling was in accordance with the NIOSH-2016 method. FA inhalation within the indoor air of the anatomy dissection room was collected using silica gel-coated sorbent tubes connected to personal air sampler pumps. The flow rate of the samples was maintained at 0.1 L/min for a four-hour period at each location. Detection and quantification were facilitated by an ultraviolet detector equipped with a stainless-steel column and packed with 5 μm C-18. For quality control, a blank cartridge was analyzed across six levels of known FA concentrations (ranging from 0.01 to 10 ppm), yielding correlation coefficients exceeding 0.97. The analytical data obtained were rigorously adjusted by subtracting the values attributed to the blank sample.

### Culture of fungi and bacteria for DNA extraction

The swab method was used to collect microbial samples from the surface of cadavers. Two sampling sites, designated ANA03 and ANA08, were selected because they are frequently contacted by health science students. Samples were collected using sterile cotton swabs from an area of approximately 5 × 5 cm on the cadaver surface. Each sampling was performed in triplicate using separate sterile swabs for each replicate. Swabs were immediately placed in sterile 20 mL glass tubes, transported on ice, and subsequently inoculated into two different broth media, including nutrient broth (NB), which is commonly used as a simple and standard medium to culture a wide range of non-fastidious bacteria and potato dextrose broth (PDB) for growing fungi. The nutrient broth containing the inoculated bacterial cells was shaken at 37 ± 2°C at a speed of 150 rpm for the three-day incubation period. Fungi were grown in a potato dextrose broth for five days at 30 ± 2°C and a shaking speed of 150 rpm. The bacterial and fungal cell cultures grown from the broth were then utilized for cell enumeration and DNA extraction to determine the quantities and communities they belonged to.

### DNA extraction, sequencing, and bioinformatics analysis

ANA03 and ANA08 cultures obtained from the previous section were used for DNA extraction to analyze bacterial and fungal community structures. Genomic DNA was extracted using the PowerSoil® DNA Isolation Kit (Mo Bio Laboratories, USA) from biomass grown in NA and PDA broths. The V3–V4 region of the 16S rRNA gene was amplified by PCR and sequenced on the MiSeq platform (Illumina Inc., USA) to assess bacterial diversity, while fungal diversity was analyzed using ITS1–2 amplicon primers. In preparation for the amplicon sequence analysis, the obtained sequences were sorted and qualified using the Quantitative Insights into Microbial Ecology (QIIME2) Pipeline, Version 2022.2 [14]. q2- CUTADAPT was utilized to remove the forward and reverse primer sequences (19 and 20 bps, respectively) [15]. For error correction on the acquired sequences utilizing amplicon sequence variation, the DADA2 package was utilized (ASV) [16]. The identified ASVs were classified taxonomically using the SILVA database version 138 [17]. The ASVs were classified taxonomically using the Naive Bayes classifier [18]. A phylogenetic assessment of bacterial communities was undertaken by reconstructing unobserved states for the function prediction investigation (PICRUSt) [19]. After receiving KEGG Orthology (KO) data, the abundances of diverse functionally relevant enzymes in bacterial communities were evaluated. The Illumina MiSeq raw sequencing data have been submitted to the NCBI Sequence Read Archive (SRA) and are available under BioProject accession number PRJNA1337940.

### Statistical analysis

Data were analyzed using IBM SPSS Statistics Version 23.0. FA concentrations were summarized as mean ± standard deviation (SD), and TWA values were calculated for both personal and indoor air samples during each dissection session. Differences in FA concentrations between anatomical regions and dissection depth levels were compared using the Kruskal-Wallis test. If a significant difference was found, pairwise comparisons were performed using the Mann-Whitney U test. To explore the relationship between dissection depth and FA concentration, Spearman’s rank correlation was used. A p-value of less than 0.05 was considered statistically significant.

## Results

### FA concentration results

This study was conducted in a naturally ventilated gross anatomy laboratory located within an Am climate, where FA exposure levels were systematically assessed. The analysis included both personal exposure measurements and ambient indoor air concentrations, providing a comprehensive view of FA dynamics in the dissection environment.

**Fig 2:**
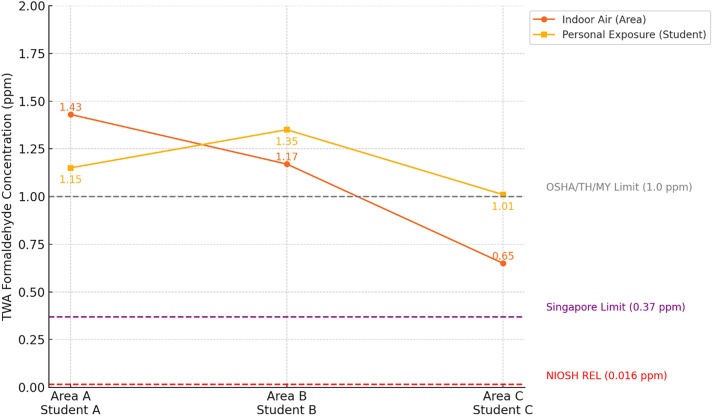
TWA formaldehyde concentrations (ppm) at paired indoor air (Area A–C) and personal exposure (Student A–C) points, shown with reference limits from NIOSH, Singapore, and OSHA/Thailand/Malaysia. NIOSH REL; NIOSH Recommended Exposure Limit, OSHA; Occupational Safety and Health Administration, MY; Malaysia, TH; Thailand.

**Fig 3:**
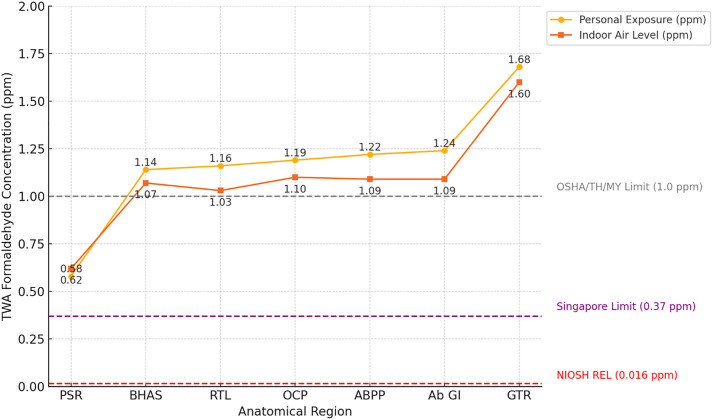
TWA formaldehyde concentrations (ppm) by anatomical region, based on indoor air and personal exposure, compared with NIOSH, Singapore, and OSHA/Thailand/Malaysia exposure limits. NIOSH REL; NIOSH Recommended Exposure Limit, OSHA; Occupational Safety and Health Administration, MY; Malaysia, TH; Thailand.

The TWA concentration of personal FA exposure among students was 1.17 ± 0.39 ppm, with values ranging from 0.22 to 1.68 ppm (Table 1). Student B exhibited the highest mean exposure (1.35 ± 0.50 ppm), followed by Student A (1.15 ± 0.23 ppm) and Student C (1.01 ± 0.38 ppm). Although visual inspection suggested elevated exposure during specific dissection stages, a Kruskal-Wallis test revealed no statistically significant difference in personal FA exposure across anatomical regions (H(6) = 10.62, p = 0.101). Nonetheless, the GTR recorded the highest individual exposure (1.68 ± 0.01 ppm), with other high-exposure regions including the AbGI and ABPP. These concentrations substantially exceeded occupational exposure limits established by NIOSH (0.016 ppm) and Singapore (0.37 ppm) (Figs 2, 3).

**Table 1:** An eight-hour time-weighted average FA exposure of personal and indoor air sampling.

Sample	Eight-hour time-weighted average (TWA) FA concentration (ppm)							
	Anatomical regions							Total (Mean±SD)
	PSR	BHAS	RTL	OCP	ABPP	AbGI	GTR	
**Personal**								
Student A	1.15	1.03	1.04	1.02	1.07	1.10	1.67	1.15 ± 0.23
Student B	0.22	1.40	1.49	1.53	1.55	1.57	1.68	1.35 ± 0.50
Student C	0.37	0.99	0.95	1.03	1.04	1.05	1.67	1.01 ± 0.38
**total** (Mean±SD)	0.58 ± 0.49	1.14 ± 0.22	1.16 ± 0.29	1.19 ± 0.29	1.22 ± 0.29	1.24 ± 0.29	1.68 ± 0.01	1.17 ± 0.39
Kruskal-Wallis p-value	0.101							
**Indoor**								
Area A	0.74	1.49	1.43	1.51	1.52	1.55	1.79	1.43 ± 0.33
Area B	0.71	1.18	1.12	1.23	1.20	1.18	1.56	1.17 ± 0.25
Area C	0.41	0.55	0.54	0.55	0.53	0.55	1.45	0.65 ± 0.35
**total** (Mean±SD)	0.62 ± 0.18	1.07 ± 0.48	1.03 ± 0.45	1.10 ± 0.49	1.09 ± 0.51	1.09 ± 0.50	1.60 ± 0.18	1.09 ± 0.45
Kruskal-Wallis p-value	0.268							

PSR; Pectoral and scapular region, BHAS; Bisection of head and air sinuses, RTL; Respiratory tract and lung, OCP; Oral cavity and pharynx, ABPP; Anterior abdominal wall, peritoneum, and peritoneal cavity, AbGI; Abdominal viscera and removal of the GI tract, GTR; Gluteal and thigh regions.

Indoor air sampling yielded a mean TWA concentration of 1.09 ± 0.45 ppm, with values ranging from 0.41 to 1.79 ppm (Table 1). Among the three measurement locations, Area A exhibited the highest concentration (1.43 ± 0.33 ppm), followed by Area B (1.17 ± 0.25 ppm), and Area C (0.65 ± 0.35 ppm). All sampling points exceeded NIOSH and Singaporean permissible exposure limits; however, Area C remained below the 1.0 ppm threshold recommended by Occupational Safety and Health Administration (OSHA), Thailand, and Malaysia (Fig 3). A Kruskal-Wallis test assessing indoor air concentrations across anatomical regions also showed no statistically significant differences (H (6) = 7.61, p = 0.268). Although not statistically significant, indoor FA concentrations varied in relation to the anatomical regions dissected. The highest level was recorded during the GTR stage (1.60 ± 0.18 ppm), which involved dissection of deep musculature and connective tissues. Elevated concentrations were also observed during the ABPP (1.09 ± 0.51 ppm) and AbGI (1.09 ± 0.50 ppm) stages, both of which involved deep abdominal regions with thick fat layers. In contrast, the lowest concentration was recorded during the PSR stage (0.62 ± 0.18 ppm), which focused on more superficial anatomical structures. Intermediate levels were observed during BHAS (1.07 ± 0.48 ppm), RTL (1.03 ± 0.45 ppm), and OCP (1.10 ± 0.49 ppm). While these values show some variability, dissection stages involving deeper or denser tissues generally corresponded with higher FA concentrations. As with personal exposure, all indoor concentrations across regions exceeded NIOSH and Singapore standards, with PSR being the only stage remaining below the OSHA, Thailand, and Malaysia limits (Fig 3).

FA concentrations from both personal and indoor air samples demonstrated variation according to the depth of the anatomical dissection regions (Table 2). For personal exposure, the mean FA levels increased progressively with dissection depth: superficial (0.86 ± 0.47 ppm), intermediate (1.18 ± 0.26 ppm), and deep (1.38 ± 0.36 ppm). The Kruskal–Wallis test indicated a statistically significant difference across the three depth categories (p = 0.020). Pairwise comparisons using Mann–Whitney U tests showed a significant difference between intermediate and deep regions (p = 0.013), whereas differences between superficial and other levels were not statistically significant. Indoor FA concentrations followed a similar increasing trend by depth but did not reach statistical significance (superficial: 0.78 ± 0.17 ppm; intermediate: 1.07 ± 0.45 ppm; deep: 1.40 ± 0.32 ppm; p = 0.121). Spearman’s rank correlation further supported the association between depth and FA levels, revealing a significant positive correlation for both personal (ρ = 0.623, p = 0.003) and indoor measurements (ρ = 0.445, p = 0.043).

**Table 2:** Comparison of personal and indoor FA concentrations by dissection depth.

Depth Level	Personal FA (Mean ± SD, ppm)	Indoor FA (Mean ± SD, ppm)
Superficial	0.86 ± 0.47	0.78 ± 0.17
Intermediate	1.18 ± 0.26	1.07 ± 0.45
Deep	1.38 ± 0.36	1.40 ± 0.32
**Kruskal-Wallis** (p-value)	0.020^*^	0.121
**Mann-Whitney U** (p-value)		
Superficial vs Intermediate	0.165	–
Superficial vs Deep	0.078	–
Intermediate vs Deep	0.013^*^	–
**Spearman’s rho** (ρ)	0.623	0.445
**Spearman** (p-value)	0.003^*^	0.043^*^

*p < 0.05; ρ = Spearman’s rank correlation.

### Bacterial community structure analysis

**Fig 4:**
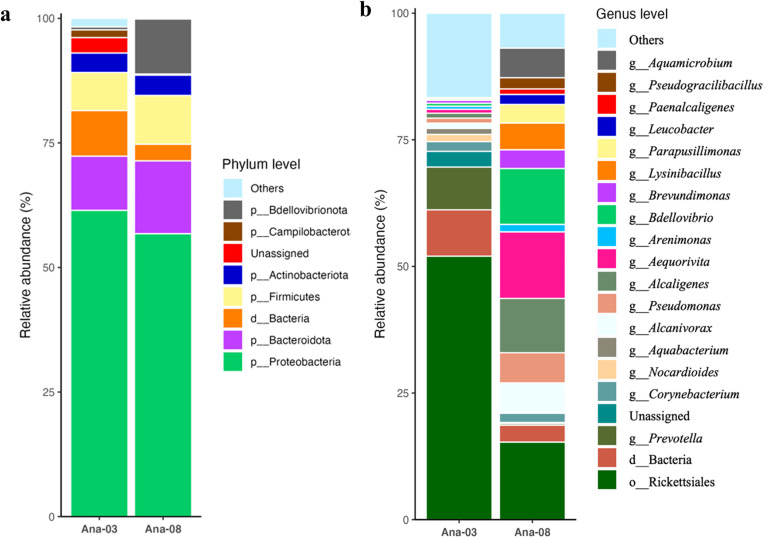
Abundances of different types of bacteria at the phylum (a) and genus (b) levels were related to the cultured bacterial cells obtained from the cadavers.

The ANA03 and ANA08 were chosen as sample sites because they are usually contacted by health science students. 16S rRNA sequencing was employed to precisely characterize bacterial populations obtained from the cadaver swab specimen. In addition, the bacteria were reported to have grown fast throughout 2–3 days of swabbing and incubation in nutrient broth. Upon analysis for diversity, samples ANA03 and ANA08 displayed significant distinctions. In particular, sample ANA08 displayed the greatest diversity of bacterial species (Fig 4a and b). In these bacterial communities, the majority of the sequences were assigned to *Proteobacteria* (ANA03; 61.50% and ANA08; 56.70%), *Bacteroidota* (ANA03; 10.90% and ANA08; 14.60%) and *Firmicutes* (ANA03; 7.60% and ANA08; 9.80%). Interestingly, in ANA08 sample, *Bdellovibrionota* was found, accounting for 11.05% (Fig 4a). The dominance of this phylum corresponds to the relative abundance of the genus *Bdellovibrio* (Fig 4b), which increased its abundance to 11.05% compared to 0.59% for ANA03.

Fig 4b illustrates the significant relative abundance of bacterial communities at the genus level for ANA03 and ANA08. The diversity of other genera varied between the two samples, showing noticeable differences in their abundance. The major bacterial orders were largely consistent across both samples, with Rickettsiales, representing the most abundant taxon, accounting for 52.02% in ANA03 and 15.31% in ANA08. Due to the limited resolution of the 16S rRNA gene sequences in distinguishing closely related lineages within Rickettsiales, the classification could not be reliably assigned to the genus level; therefore, we report this taxon at the order level. Additionally, the observed abundance of *Bdellovibrio* was found to be less than 3.40 times more abundant in ANA08 compared to ANA03. This suggests that the environment may favor the growth of a bacterial pathogen.

Although phenol-glycerin solutions used in cadaver preparation possess antimicrobial properties, contamination may still occur due to the presence of infected bacterial cells. In sample ANA08, the bacterial community was dominated by *Aequorivita* (13.13%), *Alcaligenes* (10.73%), *Pseudomonas* (5.95%), and *Alcanivorax* (5.81%) in ANA08. In contrast, sample ANA03 exhibited a broader diversity of genera with lower individual abundance. *Prevotella* was the most prevalent (8.45%), followed by *Rickettsiales*, a well-known pathogenic group.

### Fungal community structure analysis

**Fig 5:**
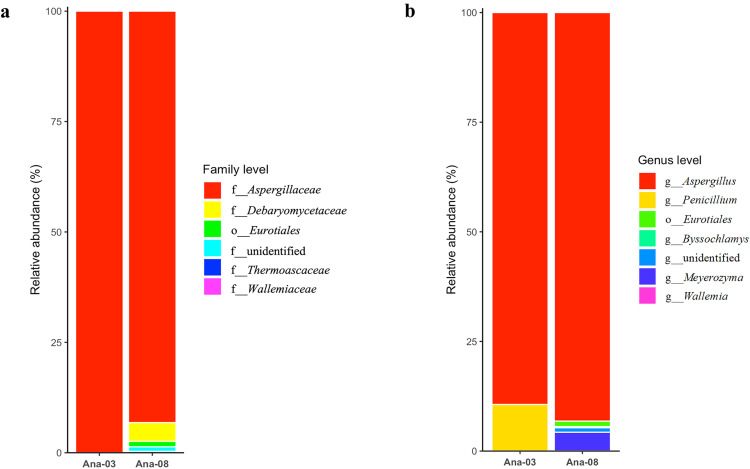
Abundances of different types of fungi at the family (a) and genus (b) levels were related to the cultured fungi cells obtained from the cadavers.

The relative abundance of fungi according to classification family and genus is shown in Fig 5a and b. The bacterial composition of ANA03 and ANA08 was found to be low in diversity, comprising a total of 2 bacterial phyla (*Ascomycota* and *Basidiomycota*). At the family level, the ANA03 fungal community was entirely comprised of *Aspergillaceae*, whereas the ANA08 community was composed of *Aspergillaceae*, *Debaryomycetaceae*, *Thermoascaceae, Wallemiaceae* and unclassified order *Eurotiales* and unclassified culture. *Aspergillaceae* dominated ANA08 with a 93.17% abundance, followed by the other family, as shown in Fig 5a. This corresponded with the relative abundance of *Aspergillus* spp. was 89.40% and 93.20% in ANA03 and ANA08, respectively (Fig 5b). In addition, the relative abundance of *Penicillium* was 10.60%, but fungi in this genus were not detected in the ANA08 sample. It could be concluded that *Aspergillus* was the main functional fungus involved in fungal contamination in samples ANA03 and ANA08.

The ANA08 was a complex microbial mix composed of various genera, as illustrated in Fig 5b. Given that *Meyerozyma was* among the abundant yeasts in the ANA08, which might be potentially serious, especially for opportunistic pathogens, and that it has been isolated from several human illnesses [20,21]. *Byssochlamys* was one of the diverse fungal genera detected in ANA08. The genus includes five species forming sexual structures (*B. fulva*, *B. lagunculariae*, *B. nivea*, *B. spectabilis*, *B. zollerniae*) [22].

### Functional prediction of pathogen and chemical-degrading bacterial populations using PICRUSt2

**Fig 6:**
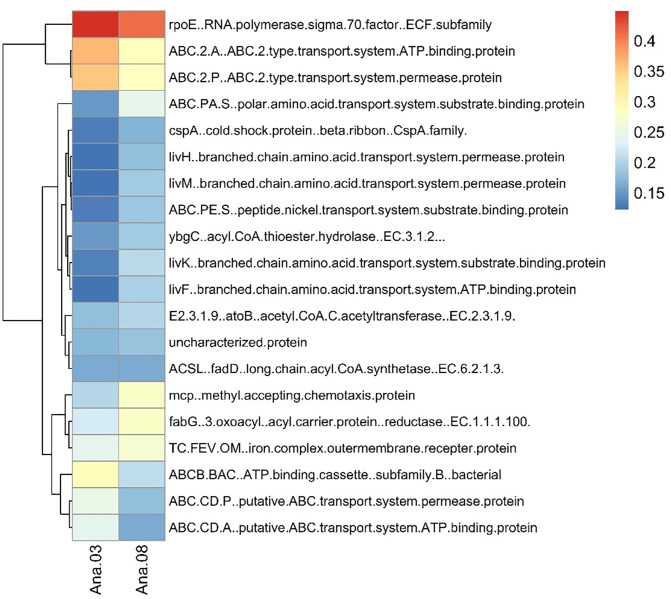
Predicted bacterial functional profiles of the relative abundances of the top 20 functions in ANA03 and ANA08 using PICRUSt2.

Fig 6 presents a heatmap comparing the relative abundance of predicted functional genes between samples ANA03 and ANA08, based on high-throughput sequencing and functional annotation. The color gradient represents normalized abundance levels, ranging from 0.15 (blue) to 0.4 (red). Genes involved in various transport systems, metabolic processes, and regulatory functions are shown clustered according to similarity in their expression profiles.

In sample ANA08, there is a notably higher abundance of genes associated with ABC transporters, such as *ABC.2.A* (ATP-binding protein), *ABC.2.P* (permease protein), and *ABC.PA.S* (substrate-binding protein), suggesting an enhanced capability for transporting a wide range of substrates, including hydrophobic compounds like aromatic hydrocarbons. Additionally, *rpoE*, a sigma factor involved in the extracytoplasmic stress response, and *cspA*, a cold shock protein, are also elevated in ANA08, indicating microbial adaptation to environmental stress, possibly triggered by exposure to xenobiotic compounds such as polyaromatic hydrocarbons (PAHs). Genes encoding enzymes involved in branched-chain amino acid and peptide transport systems (*livH*, *livM*, *livF*) are also enriched in ANA08. In contrast, ANA03 exhibited a more diverse but lower abundance of these functional genes. Collectively, these findings suggest that the microbial community in ANA08 is functionally more equipped for the degradation and transport of aromatic compounds, potentially reflecting microbial adaptation to a chemically enriched or contaminated cadaveric environment.

The functional profiles of the microbial communities in ANA03 and ANA08 exhibited key enzymes involved in aromatic compound degradation. Notably, *phenol 2-monooxygenase* (EC 1.14.13.7) and *catechol 1,2-dioxygenase* (*catA*, EC 1.13.11.1) were responsible for the aerobic breakdown of phenol and catechol [23]. These enzymes facilitated the conversion of toxic aromatic compounds into metabolizable intermediates, enabling microbial survival in chemically stressed environments. The variation in their relative abundance between ANA03 and ANA08 suggests distinct microbial capacities to degrade and tolerate phenolic compounds, reflecting potential adaptation to differing levels of chemical exposure.

## Discussion

### FA exposure in an Am environment: Concentration patterns and variability

This study identified substantial occupational exposure to FA in a gross anatomy dissection hall located in an Am climate and operating under natural ventilation. Based on TWA measurements, the mean personal exposure level among students was 1.17 ± 0.39 ppm, with individual values ranging from 0.22 to 1.68 ppm. These values exceeded multiple international safety standards, including the NIOSH recommended exposure limit (REL) of 0.016 ppm and Singapore’s permissible exposure limit (PEL) of 0.37 ppm. The overall mean indoor TWA concentration was 1.09 ± 0.45 ppm, with Area A showing the highest level (1.43 ± 0.33 ppm) and Area C the lowest (0.65 ± 0.35 ppm). In Area C, levels remained within the 1.0 ppm threshold established by OSHA, Thailand, and Malaysia. However, concentrations in the remaining zones exceeded permissible limits. This finding highlights the limitations of natural ventilation in warm, high-humidity environments.

These elevated levels likely result from both limited air exchange and the climatic properties of Am environments. Although environmental parameters such as humidity, temperature, and airflow velocity were not directly recorded, a limitation of this study existing experimental literature provides clear support for their impact. Parthasarathy et al. [4] found that a 10 °C increase in temperature could double the FA emission rate in indoor environments, particularly under humid conditions. FA concentrations increased exponentially with temperature and humidity [4,24], while Liang et al. [25] reported seasonal surges up to 20-fold under warm, moist conditions. During the study period (August-September 2023), meteorological data from the NASA POWER database confirmed persistently humid conditions in Nakhon Si Thammarat (mean temperature ≈ 27 °C; relative humidity ≈ 84%; wind speed ≈ 9 m/s), consistent with the Am climate [26]. These findings suggest that the elevated concentrations in our setting were likely amplified by climatic factors inherent to the region.

Beyond general trends, marked spatial disparities in FA concentrations were evident across the dissection hall, reflecting the influence of ventilation layout and room geometry on pollutant dispersion. Area A, positioned along the room’s periphery and most distant from the exhaust outlets, exhibited the highest TWA level, whereas Area C, adjacent to the exhaust systems, showed the lowest. Marked spatial disparities in FA concentrations were influenced by ventilation layout and room geometry. Area A, located farthest from the exhaust outlets, likely experienced poor airflow, contributing to elevated levels. Handady et al. [27] confirmed higher FA concentrations in peripheral zones with limited ventilation. Similarly, Zuber et al. [28] showed that naturally ventilated dissection halls exhibit low-velocity airflow and recirculation patterns, leading to pollutant buildup in stagnant areas. These findings highlight the elevated exposure risk in under-ventilated zones. Durongphan et al. [29] further demonstrated that relocating cadaver tables from low-ventilation zones significantly reduced room-level FA concentrations, underscoring the importance of spatial design.

Interestingly, personal exposure did not always align with ambient levels. Student B recorded the highest personal TWA (1.35 ± 0.50 ppm) despite occupying a central position, highlighting the influence of proximity to cadavers and breathing-zone airflow. This supports prior observations by previous researchers, who reported that individuals performing dissections often face higher risks than ambient measures suggest [11,30,31]. Behavioral and task-based factors also contributed to variability in exposure. Students typically worked in groups of 6–10 per cadaver, but only one or two were actively engaged in dissection at any time. Those handling embalmed tissue directly and leaning over cadavers were positioned in high-exposure zones, while passive participants experienced lower risk. Dopart et al. [32] reported significant within-group exposure variability based on anatomical region and task performed. Ryan et al. [33] similarly found that short bursts of intensive dissection caused temporary but substantial exposure peaks. Mirabelli et al. [34] confirmed that working in close proximity to preserved tissue increased inhalation risk due to breathing-zone positioning. Together, these findings highlight the necessity of incorporating personal monitoring alongside area sampling to fully characterize exposure risk particularly in naturally ventilated dissection halls where airflow is heterogeneous and behavior-driven variability is substantial.

### Anatomical region as a determinant of exposure: Patterns and procedural risk differentiation

This study provides compelling evidence that FA exposure, both personal and indoor, varies substantially with the anatomical region dissected. GTR yielded the highest personal TWA (1.68 ± 0.01 ppm) and indoor air concentrations (1.60 ± 0.18 ppm), while the PSR showed the lowest levels (0.58 ± 0.49 ppm and 0.62 ± 0.18 ppm, respectively). These differences are attributable to the anatomical and procedural complexity of each region. Regions such as the AbGI and ABPP involve prolonged manipulation and sustained proximity to preserved tissues, contributing to increased FA release. High-adipose regions like the gluteal area are known to retain and slowly emit FA, elevating local concentrations during dissection. These findings are consistent with prior reports. Klein et al. [35], observed heightened FA exposure during thoracic and abdominal dissections, emphasizing the need for targeted ventilation. Vohra [31] highlighted how procedural proximity and tissue type influence personal FA levels, while Keil et al. [2] identified internal cavities and fat-rich regions as primary contributors to peak emissions.

To quantify procedural intensity, dissection regions were further classified by depth. Personal FA concentrations exhibited a stepwise increase: Superficial (0.86 ppm), Intermediate (1.18 ppm), and Deep (1.38 ppm). The Kruskal–Wallis test confirmed a significant overall difference (p = 0.020), with post hoc analysis indicating a significant contrast between intermediate and deep levels (p = 0.013). Spearman’s correlation showed a strong positive association with personal (ρ = 0.623, p = 0.003) and indoor (ρ = 0.445, p = 0.043) exposure, indicating that deeper dissections systematically pose higher FA risk [30].

Two mechanistic factors contribute to this pattern. Chemical retention in adipose tissue plays a major role; the GTR, being fat-rich, stores large quantities of lipid-soluble formaldehyde. Tissue disruption during dissection leads to concentrated vapor release a phenomenon previously classified as high-emission during fat removal [12,36]. Reduced ventilation in enclosed cavities further amplifies exposure. When confined spaces such as the ABPP and AbGI are opened, accumulated FA is released into poorly ventilated zones, where stagnant air hinders dispersion and overwhelms local exhaust systems [11,13].

These findings validate the proposed ordinal depth classification as a practical tool for exposure risk assessment. Anatomy departments can integrate this framework to anticipate high-emission sessions and deploy targeted interventions. Specifically, enhanced localized ventilation, chemical neutralization, and tailored dissection planning should be prioritized for deep procedures involving abdominal and gluteal regions. Such approaches offer the potential to mitigate occupational exposure while preserving the instructional value of gross anatomy education.

### Comparative analysis with international studies: Highlighting the impact of climate and ventilation type

This study offers clear evidence that both climatic conditions and ventilation design play pivotal roles in shaping FA concentrations in gross anatomy laboratories. Among the international and regional studies reviewed (Table 3), our facility located in an Am climate and reliant on natural ventilation, recorded the highest levels of FA exposure: a mean personal TWA of 1.17 ± 0.39 ppm and an indoor air concentration of 1.09 ± 0.45 ppm. These values substantially exceed multiple international safety thresholds, including those established by NIOSH (0.016 ppm), Singapore (0.37 ppm), and OSHA/Thailand/Malaysia (1.0 ppm).

**Table 3:** Comparison of personal and indoor FA concentrations (modified from [30]).

Authors	Country	Köppen Climate Classification [25]	Ventilation Type	Cadaver Number	Personal Exposure Mean (range) ppm	Indoor FA Concentrations Mean (range) ppm
Ohmichi et al. [11]	Japan	Cfa	Diffuser supply + 4 ACs + 8 return grills	Not mentioned	0.75	(0.23-1.03)
Lakchayapakorn & Watchalayarn [37]	Thailand (Central)	Aw	Natural ventilation + 6 fans	Not mentioned	0.66 (0.40-0.58)	0.49 (0.40-0.58)
Vohr et al. [31]	Saudi Arabia	BWh	Air conditioning + 4 exhaust fans	14	0.75	(0.68-0.85)
Yaacob et al. [38]	Malaysia	Af	Not mentioned	Not mentioned	–	0.17
Klein et al. [35]	USA	Cfa	Ventilated dissection tables + exhaust	Not mentioned	0.10	0.05
Aung, W.-Y. et al. [30]	Myanmar	Aw	Natural ventilation + 10 fans	2–3	0.44 (0.06-1.72)	0.43
Durongphan et al. [39]	Thailand (Central)	Aw	General + AC + 54 ceiling diffusers + 81 return grilles	90	(0.67-1.94)	(0.28-0.75)
Soonklang & Saowakon [40]	Thailand (Central)	Aw	Natural ventilation + 6 fans	20	0.96	0.52
Soonklang et al. [41]	Thailand (Central)	Aw	Natural and mechanical ventilation	Not mentioned	0.96^*^ (0.90–0.99) 0.61^#^ (0.36–0.84)	–
Boonkhao et al. [42]	Thailand (North-East)	Aw	Not mentioned	20	–	0.05
Present study	Thailand (South)	Am	Natural ventilation + 3 exhaust fans + 6 fans	12	1.17 (0.22-1.68)	1.09 (0.62-1.60)

*Gasmet GT5000 Terra FTIR Gas Analyzer.

^#^Absorbent tube method.

Cfa; Humid Subtropical, Aw; Tropical Savanna, BWh; Hot Desert, Af; Tropical Rainforest, Cfb; Temperate Oceanic, Am; Tropical Monsoon.

A comparison with previous Thai studies highlights the impact of regional climate classifications. Most were conducted in tropical savanna (Aw) zones, where both environmental humidity and seasonal wet periods are less intense. For example, Lakchayapakorn and Watchalayarn [37], operating in central Thailand, reported markedly lower FA levels (0.66 ppm personal; 0.491 ppm indoor) under natural ventilation supplemented by six industrial fans. Similarly, Soonklang and Saowakon [40] and Durongphan et al. [39] reported indoor concentrations of 0.518 ppm and 0.58 ppm, respectively, despite similar or improved ventilation conditions. Furthermore, Boonkhao et al. [42], studying a gross anatomy laboratory in Thailand, reported a low 8-hour TWA indoor air concentration of 0.05 ppm. In contrast, our study in the Am zone, characterized by prolonged humidity and reduced dry periods showed significantly elevated concentrations, suggesting that climatic moisture and heat may enhance FA volatilization and inhibit effective dispersal.

More recently, Soonklang et al. [41] reported indoor FA levels of 0.96 ppm and 0.61 ppm using two distinct measurement methods Fourier transform infrared (FTIR) gas analyzer and absorbent tube, respectively. Although instrumentation differences may partly account for this variation, the values remain considerably below those observed in the present Am-climate setting. This highlights that climatic humidity and heat, rather than methodological variation alone, are likely major contributors to enhanced FA volatilization and retention.

Ventilation system configuration further contributed to exposure differentials. Our dissection hall utilized natural cross-ventilation, supported by three exhaust fans and six industrial standing fans. Despite this, FA concentrations remained well above those observed in laboratories with mechanical ventilation systems. In the United States (temperate climate), Klein et al. [35] reported indoor concentrations as low as 0.05 ppm using ventilated dissection tables and directional exhaust. Similarly, in Japan (humid subtropical climate) documented levels between 0.23–1.03 ppm using a combination of air conditioning and return grilles [11]. Even in the hot desert climate of Saudi Arabia, they maintained levels between 0.68–0.85 ppm using air conditioning with exhaust fans [31], and in Myanmar achieved lower concentrations (0.43 ppm) through improved airflow with ten industrial fans [30]. These patterns indicate a consistent trend: laboratories located in lower-humidity environments or those employing mechanical ventilation generally maintain safer FA levels even when cadaver use and teaching practices are similar. Our findings highlight a critical shortfall in exposure control within Am environments, where natural ventilation alone proves inadequate for managing FA accumulation. The combination of high humidity and passive airflow creates conditions conducive to both chemical and microbial hazards in anatomy teaching laboratories. This aligns with previous findings indicating that air-conditioned ventilation systems are significantly more effective in reducing FA concentrations and should be prioritized in laboratory design [28]. Given these risks, we strongly advocate for climate-adaptive infrastructure emphasizing mechanical ventilation, dehumidification, and targeted filtration to safeguard the health of students, faculty, and staff in expanding academic institutions within tropical regions.

### Impact of Am climate on microbial diversity in dissection halls

The Am climate appears to exert a strong influence on microbial diversity and persistence within dissection hall environments. The bacterial community analysis from cadaver swabs at ANA03 and ANA08 demonstrated site-specific differences in microbial structure, likely reflecting variations in humidity, temperature, and ventilation associated with tropical climatic conditions. Such conditions are known to favor microbial growth, particularly for moisture-tolerant taxa such as *Proteobacteria*, *Bacteroidota*, and *Firmicutes*, which dominated both sampling sites.

Notably, the higher bacterial diversity observed in ANA08 suggests a microenvironment that supports greater microbial activity, possibly due to higher surface moisture and increased human contact. The coexistence of predatory bacteria such as *Bdellovibrio* [43] and potentially pathogenic taxa like *Rickettsiales* indicates an ecologically dynamic system in which microbial competition, nutrient recycling, and potential biosafety risks coexist [44]. This aligns with prior reports that elevated temperature and humidity can promote both bacterial proliferation and predator–prey microbial interactions in confined environments [45].

The tropical climate may also contribute to enhanced biofilm formation and stress tolerance among surface-associated microorganisms. Genera such as *Pseudomonas* and *Alcaligenes*, enriched in ANA08, are known for producing extracellular polymeric substances (EPS) that protect against oxidative and chemical stress. These biofilm-mediated defenses could explain the persistence of microorganisms despite the use of phenol–glycerin embalming solutions with antimicrobial properties. Similar findings in other tropical healthcare and laboratory settings highlight the resilience of microbial biofilms under high-humidity conditions, which can reduce the efficacy of surface disinfectants [46,47].

The ability of certain fungi to tolerate both environmental extremes and preservation chemicals, supports the hypothesis that tropical conditions promote the survival of resilient fungal taxa in indoor environments, consistent with previous findings. Moreover, filamentous fungi represent key indoor microbial agents associated with respiratory diseases and allergenic responses, highlighting potential health risks in inadequately controlled dissection environments.

These results suggest that the Am climate promotes a unique ecological balance in dissection halls, characterized by high microbial diversity, biofilm resilience, and potential biosafety risks. Effective environmental management in such climates should therefore consider temperature and humidity control, airflow optimization, and targeted disinfection protocols that disrupt biofilms. Understanding microbial adaptation under tropical conditions not only informs cadaver preservation strategies but also contributes to broader insights into microbe–climate interactions in healthcare and educational environments.

Our findings suggest that the presence of *Byssochlamys* reflects a strong tolerance to both heat and chemical stress. This airborne genus produces heat-resistant ascospores capable of surviving temperatures up to 85 °C [22], which likely explains its persistence under tropical climatic conditions. High-throughput sequencing of fungal species associated with human cadavers provides valuable insights into local ecological dynamics and underscores the potential of fungi as forensic and environmental indicators. Further investigation is warranted to evaluate biological control strategies for cadaver preservation and to establish optimal storage conditions that minimize fungal proliferation.

### Limitations and future directions

This study provides foundational data on FA exposure and microbial contamination in an Am climate anatomy lab, but several limitations warrant consideration. Environmental parameters such as temperature, humidity, and airflow were not directly measured, limiting causal inference. As a single-site, cross-sectional study, generalizability is constrained, though the facility reflects conditions common in Southeast Asia. Microbial sampling was restricted to two cadaver-contact surfaces, which may not fully capture spatial variation. Additionally, no health data were collected, precluding links between exposure and clinical outcomes.

Future studies should incorporate environmental monitoring, expand sampling locations, and adopt longitudinal designs to assess seasonal or procedural variability. Evaluating mechanical ventilation, improved cadaver preparation, and biofilm-targeted disinfection will be essential for reducing chemical and biological risks. Health surveillance integration can further support evidence-based safety standards in anatomy education, especially in climate-vulnerable regions.

## Conclusion

Our findings reveal that gross anatomy dissection halls in Am climates pose elevated occupational risks due to excessive FA exposure and persistent microbial contamination. FA levels consistently exceeded recommended thresholds, particularly during dissections of deep anatomical regions, indicating that natural ventilation systems are insufficient under high humidity and temperature. Additionally, microbial analysis identified stress-tolerant and potentially pathogenic species with enhanced degradation capacity, suggesting environmental adaptation to chemical exposure. These results highlight the need for improved engineering controls, including mechanical ventilation, thermal regulation, and targeted disinfection strategies, to safeguard health in anatomy education settings. Climate-specific occupational safety standards and routine environmental monitoring should be prioritized in similar high-risk academic environments.
